# Supplementary material for: 3D Printing of a Reactive Hydrogel Bio-Ink Using a Static Mixing Tool
Source: Polymers (Basel). 2020 Aug 31;12(9):1986. doi: 10.3390/polym12091986 (PMC7564821; doi:10.3390/polym12091986)
Supplement: Supplementary file 1 [file polymers-12-01986-s001.pdf]

Supporting information. 3D printing of reactive hydrogel bioink using a static mixing tool

A

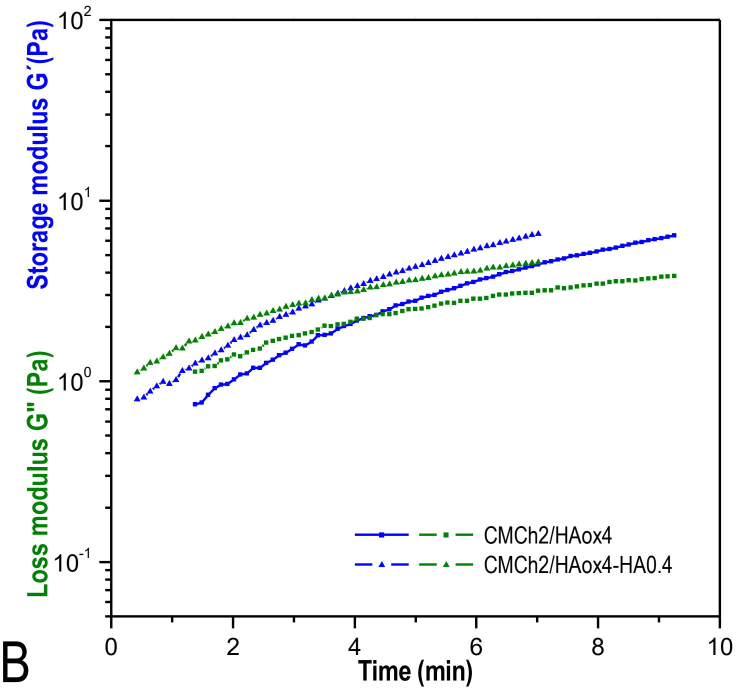

B

Formulation	CMCh2/HAox4	CMCh2HAox4-HA0.4
Gelation time/s	3.64 ± 0.43	3.65 ± 0.33

Figure S1. (A) Storage (G') and loss (G") moduli obtained in dynamic time sweep rheological experiments and (B) gelation times, defined as G' and G" crossover points, obtained for CMCh2/HAox4 and CMCh2/HAox4-HA0.4 formulations.

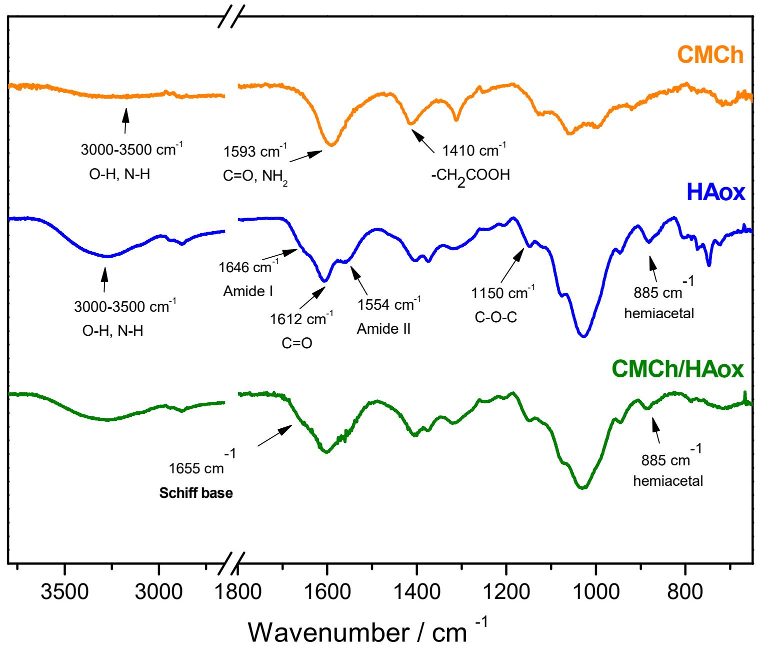

Figure S2. FTIR spectra of CMCh, HAox and CMCh/HAox reactive hydrogel.
